# Development and validation of a neural network for NAFLD diagnosis

**DOI:** 10.1038/s41598-021-99400-y

**Published:** 2021-10-12

**Authors:** Paolo Sorino, Angelo Campanella, Caterina Bonfiglio, Antonella Mirizzi, Isabella Franco, Antonella Bianco, Maria Gabriella Caruso, Giovanni Misciagna, Laura R. Aballay, Claudia Buongiorno, Rosalba Liuzzi, Anna Maria Cisternino, Maria Notarnicola, Marisa Chiloiro, Francesca Fallucchi, Giovanni Pascoschi, Alberto Rubén Osella

**Affiliations:** 1 Laboratory of Epidemiology and Biostatistics, National Institute of Gastroenterology, “S de Bellis” Research Hospital, Via Turi 27, 70013 Castellana Grotte, BA Italy; 2 Laboratory of Nutritional Biochemistry, National Institute of Gastroenterology, “S de Bellis” Research Hospital, Via Turi 27, 70013 Castellana Grotte, BA Italy; 3 Scientific and Ethical Committee, Polyclinic Hospital, University of Bari, Piazza Giulio Cesare, 11, 70124 Bari, BA Italy; 4 Human Nutrition Research Center (CenINH), School of Nutrition, Faculty of Medical Sciences, Universidad Nacional de Córdoba, Córdoba, Argentina; 5 Clinical Nutrition Outpatient Clinic, National Institute of Gastroenterology, “S de Bellis” Research Hospital, Via Turi 27, 70013 Castellana Grotte, BA Italy; 6 San Giacomo Hospital, Largo S. Veneziani, 21, 70043 Monopoli, BA Italy; 7 Department of Engineering Sciences, Guglielmo Marconi University, Via plinio 44, 00193 Rome, Italy; 8 Department of Electrical and Information Engineering, Polytechnic of Bari, Via Re David, 200, 70125 Bari, BA Italy

**Keywords:** Computational models, Machine learning, Computational biology and bioinformatics, Gastroenterology, Health care

## Abstract

Non-Alcoholic Fatty Liver Disease (NAFLD) affects about 20–30% of the adult population in developed countries and is an increasingly important cause of hepatocellular carcinoma. Liver ultrasound (US) is widely used as a noninvasive method to diagnose NAFLD. However, the intensive use of US is not cost-effective and increases the burden on the healthcare system. Electronic medical records facilitate large-scale epidemiological studies and, existing NAFLD scores often require clinical and anthropometric parameters that may not be captured in those databases. Our goal was to develop and validate a simple Neural Network (NN)-based web app that could be used to predict NAFLD particularly its absence. The study included 2970 subjects; training and testing of the neural network using a train–test-split approach was done on 2869 of them. From another population consisting of 2301 subjects, a further 100 subjects were randomly extracted to test the web app. A search was made to find the best parameters for the NN and then this NN was exported for incorporation into a local web app. The percentage of accuracy, area under the ROC curve, confusion matrix, Positive (PPV) and Negative Predicted Value (NPV) values, precision, recall and f1-score were verified. After that, Explainability (XAI) was analyzed to understand the diagnostic reasoning of the NN. Finally, in the local web app, the specificity and sensitivity values were checked. The NN achieved a percentage of accuracy during testing of 77.0%, with an area under the ROC curve value of 0.82. Thus, in the web app the NN evidenced to achieve good results, with a specificity of 1.00 and sensitivity of 0.73. The described approach can be used to support NAFLD diagnosis, reducing healthcare costs. The NN-based web app is easy to apply and the required parameters are easily found in healthcare databases.

## Introduction

Non-alcoholic liver steatosis (NAFLD) is the leading cause of chronic liver disease in Western countries. This condition increases the risk of cardiovascular disease, type 2 diabetes mellitus and chronic kidney disease and leads to increased mortality^1,2^. The condition is estimated to affect about 20–30% of the adult population in developed countries^3^. NAFLD is defined as an accumulation of Triglycerides in the hepatocytes (> 5% of liver volume) of patient with low alcohol intake (< 20 g/day in women or < 30 g/day in men), diagnosed once causes due to viral infections or other specific liver diseases have been excluded^4^. NAFLD is becoming more common among adults between 40 and 60 years of age, but the disease is also seen children^5^. A meta-analysis published in 2016 reported that this disease has an average prevalence of 23.71% in Europe^6^. Population-based studies conducted in our geographical area (district of Bari, Apulia Region, Italy), estimated a prevalence of NAFLD of around 30%, males and the elderly are most commonly affected^7^.

NAFLD is strongly associated with the metabolic syndrome and is considered the hepatic manifestation of the metabolic syndrome^8^. It can manifest as pure fatty liver disease (hepato-steatosis) or as non-alcoholic steatohepatitis (NASH), an evolution of the former in which steatosis is associated with inflammation and hepatocellular damage, and with fibrogenic activation that can lead to cirrhosis and the onset of hepatocarcinoma^9^. In general it has been established that early diagnosis of cirrhosis and elimination of the cause can stop further liver damage, increase the chances of transplant success and also reduce mortality rates^10^. According to recent EASL—EASD—EASO guidelines^11^, at the individual level the gold standard for identifying steatosis in individual patients is magnetic resonance imaging (MRI), although ultrasound scanning (US) is considered a good alternative being more widely available and cheaper than MRI. In addition, for large-scale screening studies, serum biomarkers and steatosis score indices have been preferred because their easy availability and low cost has a substantial impact on the feasibility of screening. One of the best validated indexes is the Fatty Liver Index (FLI)^12^, although other anthropometric indices or measurements work together with FLI in predicting NAFLD risk^13^.

In recent years, due to the increasing prevalence of NAFLD, there has been a research trend towards identifying low cost, diagnostic methods, and Machine Learning has been acknowledged as a valuable tool. Machine Learning (ML) is a branch of artificial intelligence aimed to enable machines to operate using intelligent "learning" algorithms^14^. Using the data sets supplied, the machine is able to process them through algorithms that allow it to develop its own logic in order to perform the required function or task. Machine Learning has already been used as a support tool for the diagnosis of different diseases, and for risk quantification, such as cardiovascular risk in patients with diabetes mellitus^15,16^, ischemic heart disease^17^ and tumors^18^.

Nowadays, NAFLD diagnosis is made by performing Ultrasound^19^ and MRI with lipid content quantification^20^. Besides some biochemical and/or anthropometric parameters alone or in combination are used to perform the diagnosis^21,22^. This implies to refer patients to more specialized health center with the consequent healthcare system burden^23^. Many studies have used ML for the diagnosis of NAFLD but they were predominantly focused to identify particular aspects of NAFLD such as quantification of lipid content, staging, fibrosis, etc^24–27^. and no longer simply ascertain the absence of disease, for example, in a large cohort of subjects avoiding in that way the use of non-invasive diagnostics for screening and monitoring NAFLD.

As imaging technologies such as ultrasound, magnetic resonance imaging (MRI), transient elastography (TE), and computed tomography (CT) are expensive and time consuming, they are generally impractical for most serial assessments^28^ or when large-scale population studies are considered. In addition to high cost, other limitations of imaging-based diagnosis of liver damage such as operator dependence, lower sensitivity and range, radiation exposure and limited availability need to be considered^29^. Moreover, ML-based models have also been used to classify liver diseases into distinct categories with ~ 80% accuracy^30,31^, highlighting that biomarker-based diagnostic methods meet the requirements for diagnosis^32^.

Then, our purpose was to develop a simple web app which permits to perform the diagnosis of absence of NALFD with high accuracy to reduce waiting list and costs for the National Health System, as. most studies on NAFLD diagnosis are based on images or laboratory parameters that are not always available^26,33^.

The aim of our study was to develop and validate a simple Neural Network (NN), using easily available laboratory parameters which had been identified in our previous study^34^, in order to build a web app incorporating the NN, trained to apply them to identify subjects at greater risk of NAFLD to be scheduled for ultrasound assessment. We also checked the performance of the trained NN by analyzing Explainability (XAI)^35^; to evaluate its reliability and ease of use and validate the results on a randomly selected sample subset extracted from a population-based study.

In the first part of this paper the population under study the variables and formula on which the AVI parameter is built have been described, then. Next, a first analysis with the t-SNE^36^ technique was performed and then we switched to an approach based on NN to search for optimal parameters to build the NN with the parameters identified. Subsequently, the NN performance and XAI are evaluated. Finally, we illustrate the development of a simple local web app tested on a population sample.

## Methods

### Population

The subjects included in the were drawn from two different cohort studies conducted at the laboratory of Epidemiology and Biostatistics of the National Institute of Gastroenterology, Research Hospital "Saverio de Bellis" (Castellana Grotte, Bari, Italy). Subjects participating in the MICOL study and NUTRIHEP study were eligible. Details on the MICOL and NUTRIHEP study populations have been published elsewhere^7,13,37^. The MICOL study is an ongoing randomized study of subjects drawn from the electoral list of Castellana Grotte (aged ≥ 30 years) in 1985 and followed up in 1992, 2005–2006 and 2013–2016. The study included a total of 2970 out of 3000 selected subjects; 56.5% were male. By 1985, 2472 subjects had been enrolled. In 2005–2006, 1697 of the original cohort were still present. In 2005–2006 this cohort was added with a randomized sample of 1273 subjects (PANEL study) aged between 30 and 50 years, to compensate for the cohort aging^38,39^. All subjects gave prior informed written consent to participate.

All procedures were performed in accordance with the ethical standards of the institutional research committee (IRCCS Saverio de Bellis approval for research and the ethics committee for the MICOL study (DDG-CE-347/1984; DDG-CE-453/1991; DDG-CE-589/2004; DDG-CE 782/2013) and, with the Helsinki Declaration of 1964. The NUTRIHEP study was conducted at the National Institute of Gastroenterology Saverio de Bellis (Castellana Grotte, Bari, Italy) in collaboration with 12 General Practitioners (GPs) operating in Putignano (Bari, Italy). The study period was from July 2005 to January 2007. By means of systematic random sampling of 1 of every 5 procedures, a sample from the general population aged ≥ 18 years had been obtained from the General Practitioners lists. Instead, we used records from a census design, because no significant difference was found between the age-sex distribution of the general population from Putignano and the subjects listed in the general practitioners' registers. Therefore, 2550 subjects were invited to participate in the survey and, 2301 (90%) agreed. NUTRIHEP subjects were followed-up in 2015–2017 then, 951 of them were included. All subjects provided written information and consent according to the 1964 Helsinki Declaration.

The subjects participating in the MICOL and NUTRIHEP studies underwent anthropometric measurements, blood sampling and hepatic ultrasound. They were weighed wearing underwear, on an electronic scale, SECA; weight was approximated to the nearest 0.1 kg. Height was measured with a SECA wall stadiometer, approximated to the nearest 1 cm. Blood pressure (BP) measurements were performed following international guidelines^40^. The mean of 3 BP measurements was calculated.

## ML algorithm development

### Data acquisition and pre-processing

The initial database for the MICOL III trial contained 2970 subjects. The sample declined to 2869 as for 101 subjects there were no data on at least one of the values among Waist Circumference (WC), Hips (HP) (variables for the constitution of AVI), Gamma-Glutamyl Transferase (GGT), Glucose. These 2869 subjects constituted the new database used for training and testing the NN using a train-test-split approach. From the NUTRIHEP database, initially composed of 2301 subjects, we randomly extracted a further 100 subjects to constitute the validation sample.

#### Variables used

The Variables used to develop the NN were: Sex, Age, Gamma-Glutamyl Transferase (GGT), Glucose, Abdominal Volume Index (AVI)^41^ and NAFLD condition.

We have previously highlighted that the best model to detect the NAFLD condition is based on the above variables. These variables were identified starting from a sample of 27 variables and exploiting a subset selection approach in order to identify the model with fewer variables and better performance^34^. Table 1 shows the formula employed to build the AVI index.

**Table 1:** Index formula and its structure.

Reference	Name	Formula
Guerrero-Romero	Abdominal volume index (AVI)	$AVI = \frac{{\left[ {2*\left( {WC} \right)^{2} + 0,7*\left( {\frac{WC}{{HC}}} \right)^{2} } \right]}}{1000}$

*WC* waist circumference, *HC* hip circumference.

AVI is the only compound index used, and this formula is easy to compute and the component variables are easily available as they consist of anthropometric measurements.

The array composed by Sex, Age, Gamma-Glutamyl Transferase (GGT), Glucose, Abdominal Volume Index (AVI) represents the X of our algorithm and the condition of NAFLD the Y.

NAFLD diagnosis was performed using an ultrasound scanner Hitachi H21 Vision (Hitachi Medical Corporation, Tokyo, Japan). Examination of the visible liver parenchyma was performed with a 3.5 MHz transducer.

### Data exploration

**Figure 1:**
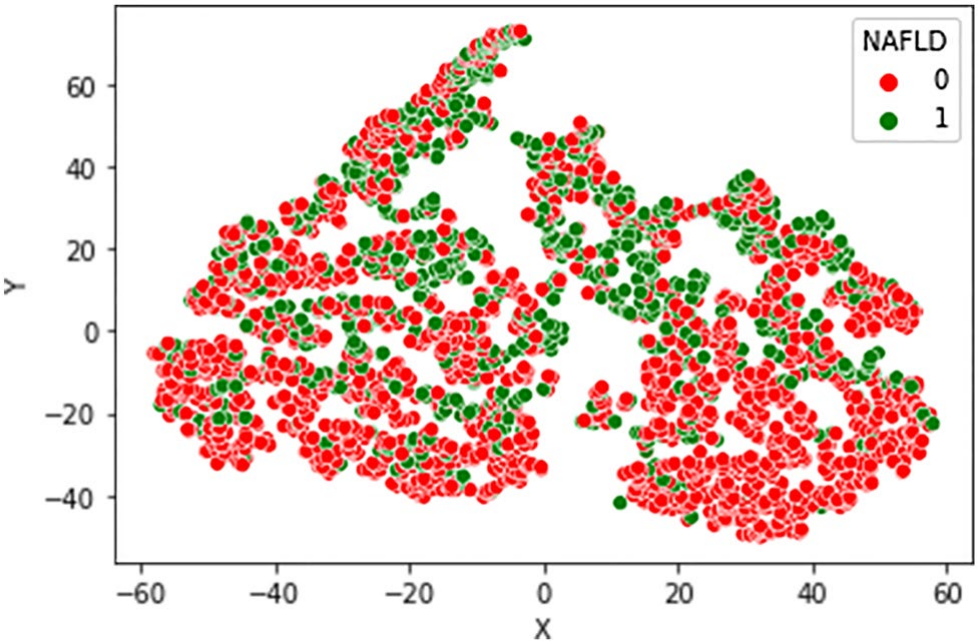
Visualizing data with t-SNE.

Data were explored by using a t-Distributed Stochastic Neighbor Embedding (t-SNE)^36^. It is an unsupervised and nonlinear technique used primarily for data exploration and visualization of high-dimensional data; its output shows how the data are organized in a high-dimensional space. This technique has not performed in optimal way failing to clearly discriminate the two classes 0 (No NAFLD), 1 (NAFLD), Fig. 1 shows data displayed with the t-SNE.

### Hyperparameter tuning for the neural network

Initially, a NN was created using the Open Source library “*scikit-learn*”^42^ by Python.

For the interaction with the csv file containing the database, the library *“numpy” (np)*^43^ by python was used.

The NN is an MLPClassifier (Perceptron Multilayer Classifier)^42^ and a supervised machine learning algorithm^44^. The first fundamental step was to split the considered database using the “*Train_test_split*” (function present in scikit-learn) in order to divide the sample into two subsets (80% of the data used for NN training and the remaining 20% for the testing).

GridSearchCV^42^ was used to search for optimal parameters for the NN.

The GridSearchCV is included in the scikit-learn library.

We have performed the NN optimization for the following parameters:

- Activation function: searched among (‘identity’, ‘logistic’, ‘tanh’, ‘relu’)
- Solver type: limited memory Broyden-Fletcher-Goldfarb-Shanno algorithm (lbfgs)^45^ Stochastic Gradient Descent^46^, Adam^47^, (‘lbfgs’, ‘sgd’, ‘adam’). "lbfgs" is an optimizer in the family of almost Newtonian methods^48^. We selected "lbfgs" because for small data sets it can converge faster and get better performance.
- Learning rate: searched among (‘constant’, ‘invscaling’, ‘adaptive’)
- the Maximum number of iterations looking for it in a defined range of values (max_iter': [1000,1100,1200,1300,1400,1500,1600,1700,1800,1900,2000, 3000,4000,5000,6000,7000, 8000,9000],) Maximum number of iterations. The solver iterates until convergence (determined by "tol") or until the maximum number of iterations.
- The alpha value searched for in a set of defined values (alpha': 10.0 ** -np.arange(0, 10),) Penalty parameter L2^49^.
- The number of hidden layers of the network 'hidden_layer_sizes': np.arange(0, 20), searched in a range from 0 to 20
- And the value of 'random_state': [0,1,2,3,4,5,6,7,8,9,10] searched in the range from 0 to 10 to make sure the results were replicable.

MLPClassifier performs iterative training because at each time step the partial derivatives^50^ of the loss function^50^ are calculated with respect to the model parameters, in order to update the parameters. It can also have a regularization term added to the loss function that reduces the Model Parameters to prevent overfitting. The values obtained at the end of the NN optimization were:

- activation: 'logistic'
- alpha: 1.0
- hidden_layer_sizes: 19
- learning_rate: 'constant'
- max_iter: 9000
- random_state: 10
- solver: 'lbfgs'

### Training session and neural network test

The algorithm was trained using as target variable the NAFLD condition and as features Sex, Age, GGT, Glucose and AVI values.

The dataset used for the training and the test of the algorithms was the MICOL subjects, subdivided into the Test and Training subsets: 80% of the dataset was dedicated to the training phase while the remaining 20% was used in the model testing phase. The output reported the accuracy during training and testing, the value of the area of the Roc curve (AUC)^51,52^ in the training and testing phase, the Confusion Matrix^53^ and the value of Precision, Recall and F1-score in the testing phase.

## Results

Participants characteristics and the performance of AVI indexes in MICOL subjects are shown in Table 2. The NAFLD prevalence was 31.7%, the condition being, as expected, more prevalent among men. Subjects with NAFLD were a little older, with increased levels of Glucose and GGT.

**Table 2:** Sample subset characteristics by NAFLD condition.

Variables	NAFLD		*p*-value
	Absent	Present	
N (%)	1961 (68.3)	908 (31.7)	
**Sex**			
Female	968 (49.4)	278 (30.6)	< 0.001
Male	993 (50.6)	630 (69.4)	
Age	53.80 (15.37)	55.00 (13.43)	0.042
AVI	15.96 (4.25)	21.17 (4.99)	< 0.001
GLUCOSE	105.27 (23.31)	117.34 (32.75)	< 0.001
GGT	14.63 (15.29)	20.63 (19.93)	< 0.001

MICOL study, Castellana Grotte (BA), Italy, 2005.

Cells reporting subject characteristics contain mean (± SD) or n (%).

In Table 3 are shown Participants characteristics and the performance of AVI indexes in the NUTRIHEP study. In the original study NAFLD prevalence was 24.3% and, as expected, more prevalent among men.

**Table 3:** Sample subset characteristics by NAFLD condition.

Variables	NAFLD		*p*-value
	Absent	Present	
N (%)	487 (51.2)	464 (48.8)	
**Sex**			
Female	298 (61.3)	231 (49.8)	< 0.001
Male	188 (38.7)	233 (50.2)	
Age	49.02 (13.42)	59.39 (13.11)	< 0.001
AVI	14.01 (3.66)	23.53 (88.04)	0.017
GLUCOSE	90.20 (10.08)	100.54 (20.86)	< 0.001
GGT	15.29 (8.18)	19.99 (15.26)	< 0.001

NUTRIHEP study, Castellana Grotte (BA), Italy, 2015.

Cells reporting subject characteristics contain mean (± SD) or n (%).

### Neural network performance analysis

The first parameter considered to evaluate the performance of the NN was the accuracy defined as^54^:

$$ {\text{Accuracy }} = { }\frac{{{\text{Number}}\;{\text{of}}\;{\text{correct}}\;{\text{preditions}}}}{{{\text{Total}}\;{\text{number}}\;{\text{of}}\;{\text{preditcions}}}}*{ }100{ } $$

More specifically, the accuracy of a model is calculated with the following formula^54^:

$$ {\text{Accuracy }} = \frac{{{\text{TP }} + {\text{ TN}}}}{{{\text{TP}} + {\text{TN}} + {\text{FP}} + {\text{FN}}}}*100{\text{\% }} $$

where TP = True Positive, TN = True Negative, FP = False Positive and FN = False Negative.

Accuracy was measured during both the NN training and the testing phase.

Another performance index that we considered was the value of the ROC curve^52^. The area under the ROC (AUC, "Area Under the Curve") is a measure of accuracy and indicates the diagnostic power of the test.

**Figure 2:**
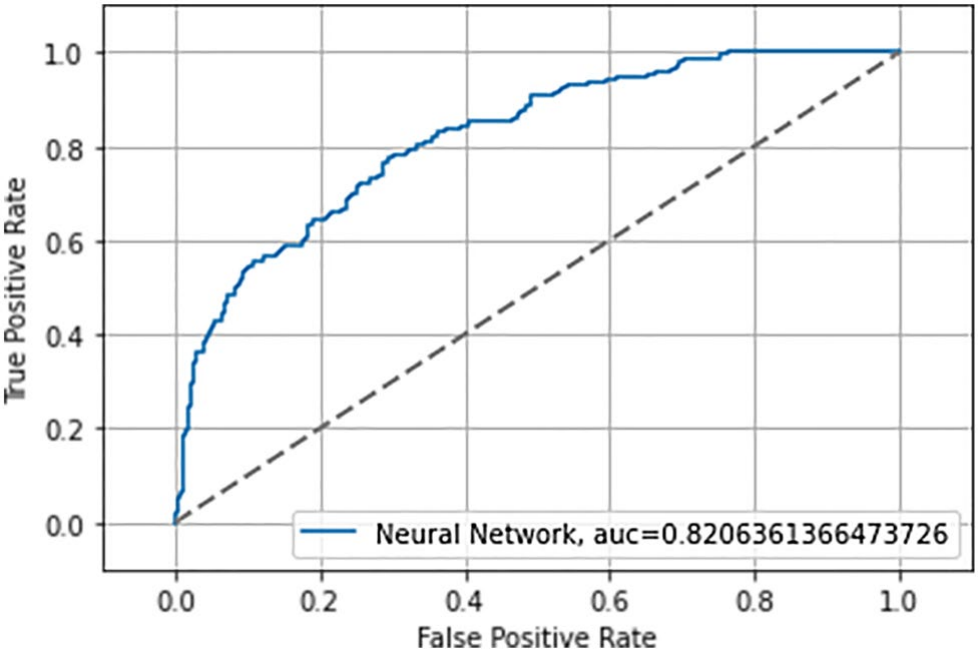
ROC curve and AUC neural network in training.

**Figure 3:**
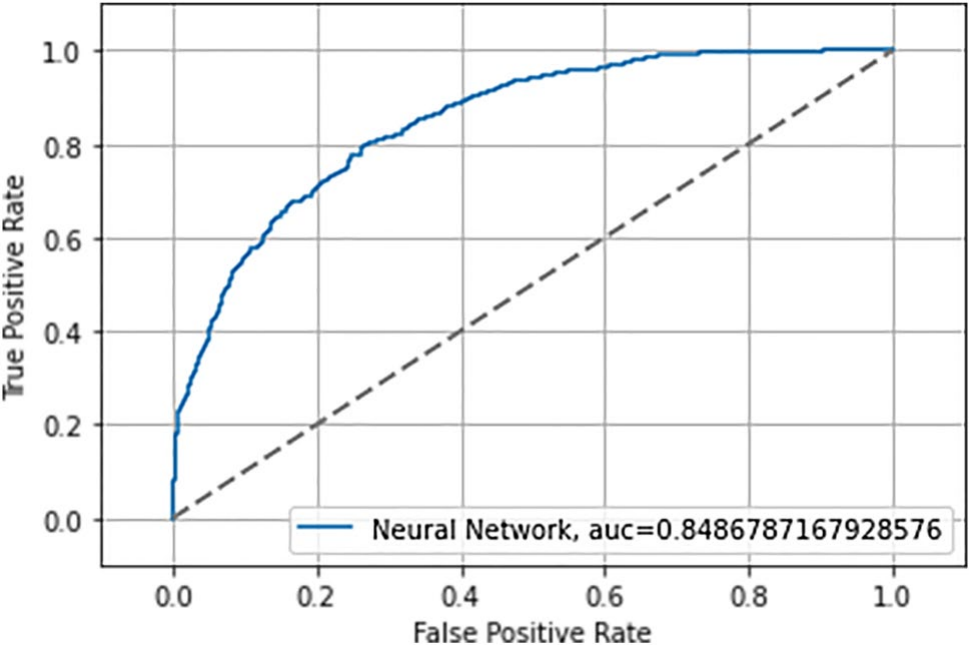
ROC curve and AUC neural network in test phase.

In Figs. 2 and 3 the ROC curves with the AUC value obtained during the training phase and testing phase are shown.

**Figure 4:**
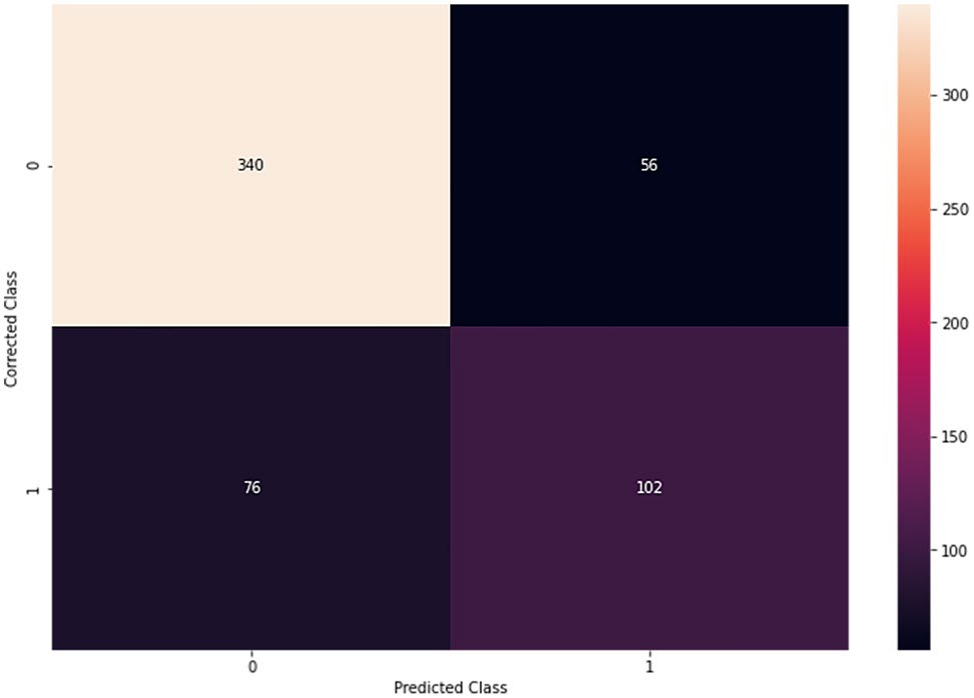
Confusion matrix values during the test. 0 indicates no presence of NAFLD, 1 indicates presence of NAFLD.

In addition to the accuracy and ROC curve values, we evaluated the confusion matrix to verify the reliability of the NN. Figure 4 shows the confusion matrix values in the test phase.

In addition, the Positive (PPV) (0.57) and Negative (NPV) (0.86) predictive values were calculated. It is worth to note that the NN is able to identify subjects without the condition with a very high precision.

Table 4 shows the Accuracy and AUC values obtained during training and testing of the NN.

**Table 4:** Accuracy and AUC values in the training and test phase.

Phase	Accuracy (%)	AUC^a^
Training NN^a^	79.1	0.84
Testing NN^a^	77.0	0.82

^a^*NN* neural network, *AUC* area under the ROC curve.

The values obtained for AUC and Accuracy (both for the training phase and for the test phase) show that the NN implemented does not present overfitting or underfitting problems, because the values of the two ROC curves and the values related to the accuracy differ very slightly. Additionally, in order to validate the performance of the NN precision, recall and f1-score values during the test phase were evaluated. In Table 5 are shown values of Precision, Recall, f1-score of No NAFLD and, NAFLD subject, Macro average and Weighted average during test phase.

**Table 5:** Value of precision, recall and F1-score on test set.

	Precision	Recall	f1-score	Support
No NAFLD	0.86	0.82	0.84	416
NAFLD	0.57	0.65	0.61	158
Macro avg^a^	0.72	0.73	0.72	574
Weighted avg^a^	0.78	0.77	0.77	574

^a^*avg* average.

### Evaluating Explainability using SHAP

**Figure 5:**
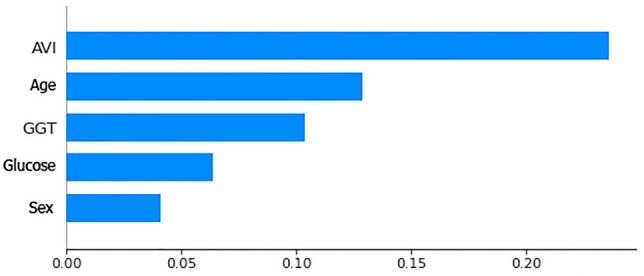
Histogram of feature relevance of anthropometric and biochemical parameters considered during training.

After verifying the behavior of the NN by comparing the various indices considered, we performed with the analysis of Explainability (XAI) using LIME^55^ and the SHAP^56^ library of Python to compare any inconsistencies. We initially proceeded to the evaluation by performing a relevance analysis of the features in order to verify whether the anthropometric and biochemical variables considered gave a real and consistent contribution in the diagnosis of NAFLD. Figures 4 and 5 show the contribution given by each feature used in the diagnosis of NAFLD within the NN during the Training and Test.

**Figure 6:**
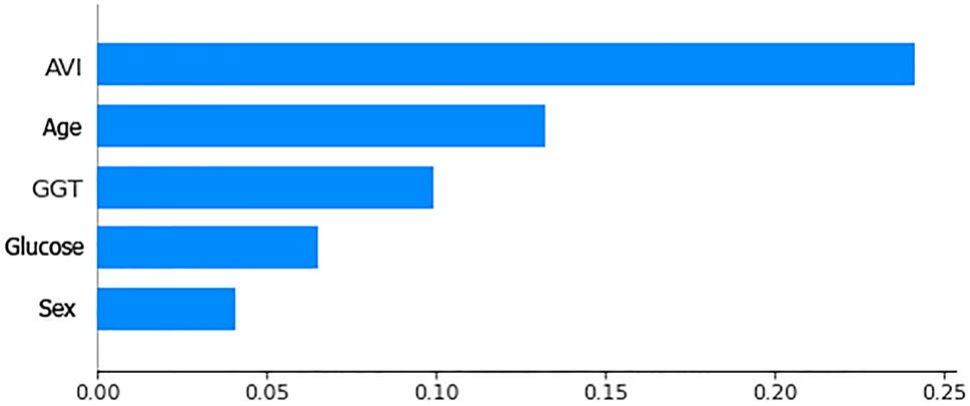
Histogram of feature relevance of anthropometric and biochemical parameters considered during test.

Figures 5 and 6 shows the importance of AVI, GGT and Age as already highlighted in previous studies^34^ are more important than sex and glucose in the diagnosis of this pathology but still combining them all together they lead to a good diagnostic result in a NAFLD diagnosis.

**Figure 7:**
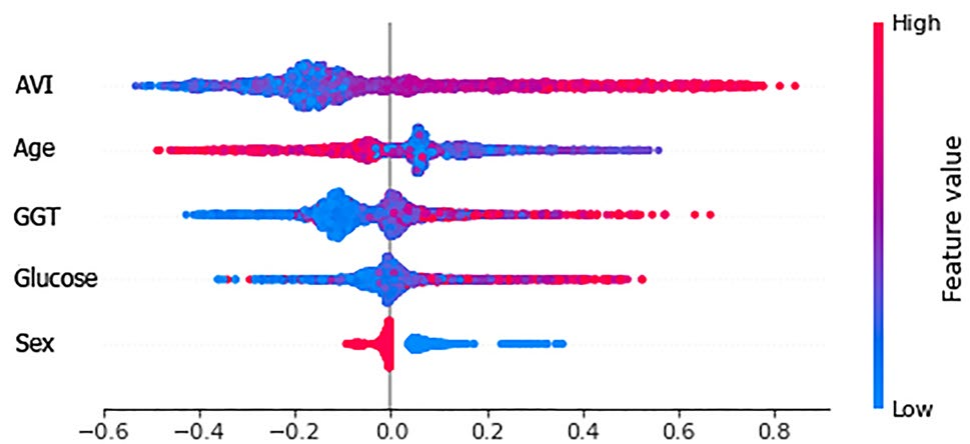
Global interpretation using Shapley values of anthropometric and biochemical parameters considered during training.

**Figure 8:**
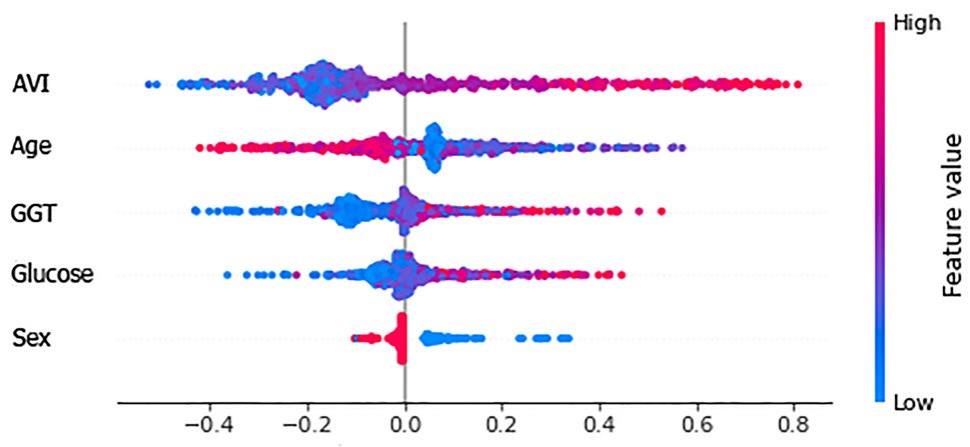
Global interpretation using Shapley values of anthropometric and biochemical parameters considered during test.

In Figs. 7 and 8 we report the previous graph seen in another way, more specifically we can understand:

- Feature importance: variables ranked in descending order of importance.
- Impact: horizontal position shows whether the effect of that value is associated with a higher or lower prediction.
- Value: color shows whether that variable is high or low for that observation. Red color deducts the high value and blue for the lower value. The change in color of the dot shows the value of the feature. Correlation: Of each characteristic with the pathology being examined.

### Evaluating Explainability using LIME

Subsequently exploiting the LIME library, it has been verified how the NN has reasoned in order to obtain a diagnosis verifying both the case of diagnosis of "sick subject" and that of "healthy subject".

**Figure 9:**
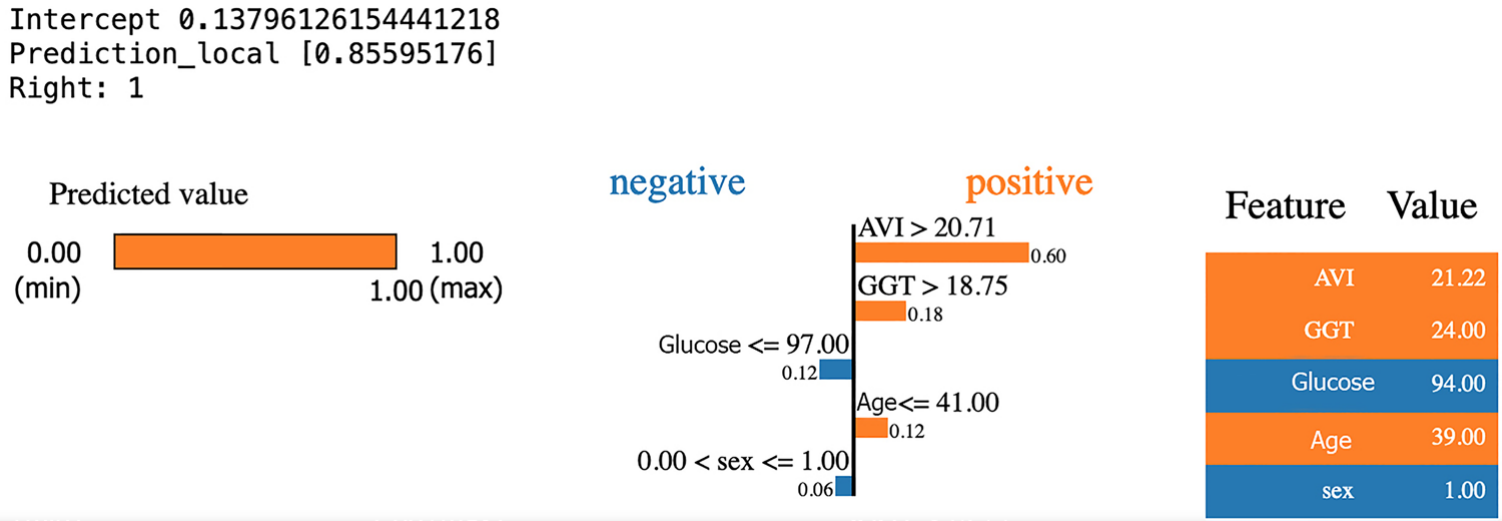
Application of LIME for diagnosis of sick subject during the test.

Figure 9 shows which characteristics had a greater impact on a diagnosis of disease present and which had a greater impact on a diagnosis of disease absent with relative final diagnosis. Regarding subjects diagnosed as sick, the features that contributed most to directing the NN toward a diagnosis of sick subject were AVI, age, and GGT value demonstrating how the NN performs optimal reasoning.

**Figure 10:**
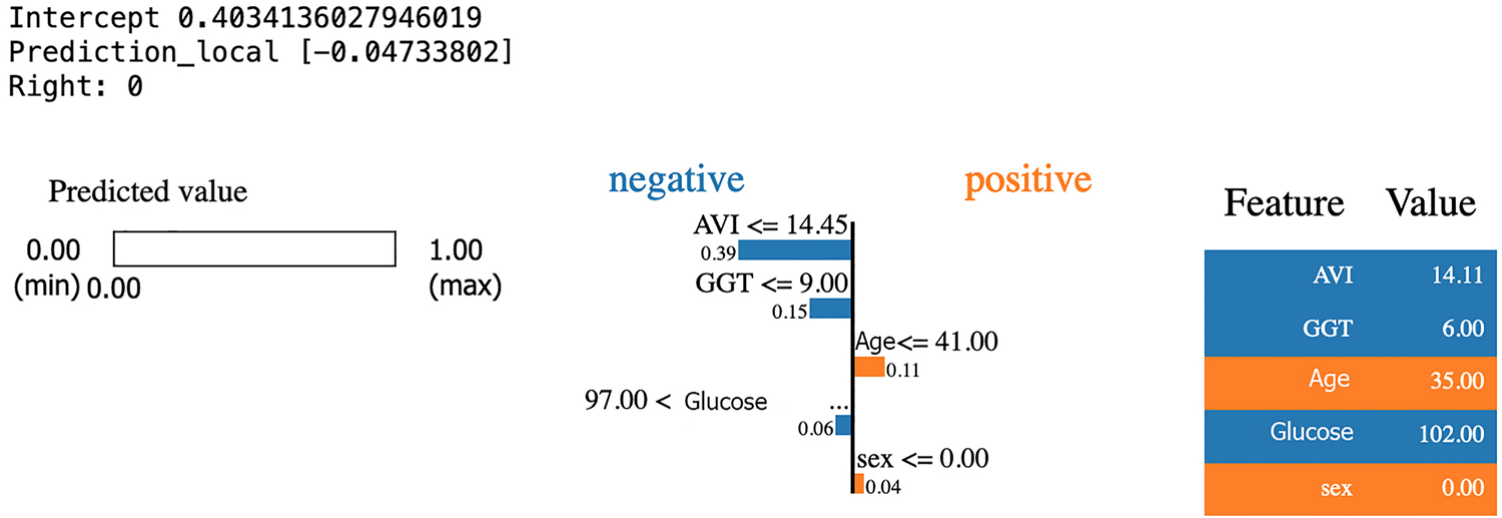
Application of LIME for diagnosis of healthy subject during the test.

Figure 10 shows what concerns the characteristics that contribute to the identification of healthy subjects, the NN took into consideration the values that from the clinical diagnosis are standard values of GGT, Glucose and a low value of the AVI index.

Also, in the diagnosis of healthy subjects the NN has produced an optimal reasoning correctly directing the diagnosis.

### Export of the trained algorithm and incorporation into the web app

After the NN training and testing and the XAI analysis we exported the already trained model. In this way it is possible to avoid repeating the training every time we want to perform a new forecast. The model export was done using the “pickle” tool by Python^57^, which allowed the generation of a file with the extension “.pkl”. This file is then loaded by means of another python program which can be used to make a new forecast. Another important function implemented is the creation of a web application written using the HTML languages^58^, CSS^59^ and JavaScript^60^. This web application can interface with the trained NN to test it on new data, different from those used to train the original NN. The interface of the web application with the trained algorithm was implemented through the “flask library”^61^ by python. A flask object receives a request from the web and displays the HTML file that allows it to interface with the NN.

The user can fill in the form present in a web page and after clicking the submit button, the flask object receives a request, extracts the input, runs it through the template and finally displays the HTML page with the result of the prediction.

The HTML page includes various fields in which to enter variables, and a submit button to pass the input data to the NN that will perform the prediction. At the end of the prediction, the HTML page will display the NAFLD status: “NAFLD Detected” or the string “No NAFLD Detected”.

The web app also includes the automatic calculation of the AVI parameter from the values for hips Circumference and waist Circumference using the code implemented in Javascript.

### Test of the web app on a sample of subjects with known NAFLD

To test the web app, the database previously formed by random extraction of 100 subjects participating in the NUTRIHEP study was used. The web app was passed the data: age, Sex, GGT, GLUCOSE, WC, HC.

After the input of the parameters and clicking the submit button, the values were sent to the NN. The web app feedback, related to the NAFLD status, was then saved in a dataset used for comparison with the true NAFLD condition, already known to us.

Using the saved dataset, we could calculate the accuracy, sensitivity and specificity of the web app.

In the sample considered, there were 50 subjects affected by NAFLD and 50 healthy subjects. The NN correctly identified all the healthy subjects but made 18 errors, all false negatives. On this result we calculated the values of Specificity and Sensitivity of the NN.

It is important to point out that many of the subjects considered healthy by the NN had anthropometric and biochemical values in the norm, but it is possible that these subjects were affected by mild NAFLD, although with values still within normal range^62^.

Table 6 shows the sensitivity and specificity values for the NN in the web app.

**Table 6:** Sensitivity and specificity values for the neural network in the web app.

Specificity	Sensitivity
1.00	0.73

## Discussion

In this study, a NN to support NAFLD diagnosis has been developed on a model made up of easily available variables, as already highlighted in our previous work^34^.

In particular, in this work we trained a NN to identify patients at risk of NAFLD and, developed a local web app for use as a tool in epidemiological studies and screening. The aim was to make a prior identification of healthy patients in order to ensure that only subjects really needing it are sent on for ultrasound examination.

Today, alternative, less expensive methods of diagnosis compared to traditional tools (MRI, Ultrasound) are very important in the diagnosis of NAFLD. The reorganization of the National Health System requires close consideration of aspects related to performance together with factors related to the reduction of costs and waiting times. The objective of our study was to create a NN implementing an intuitive and easy application to support medical decisions during the diagnostic phase using simpler and cheaper tools, thus reducing both costs and waiting times related to the use of instrumental methods. We highlight that it would thereby be possible to use simple computers to make a diagnosis of NAFLD, resulting in a faster diagnosis and thus preventing disease evolution and the resulting serious consequences.

Several prediction models for NAFLD in the literature have been developed to identify healthy subjects and subjects with NAFLD. These existing NAFLD prediction models have employed clinical and laboratory parameters; however, some parameters are not always routinely measured or retrievable in health databases^63,64^. This limits the use of these models in large-scale epidemiologic studies and health database research. Specifically comparing the AUC of NN (0.821) with traditional methods we could verify that the performance in terms of AUC is superior to LAP^65^ (0.79), Hepatic steatosis index^66^ (0.81), SteatoTest^67^ (0.79), APRI^68^ (0.60), NAFLD fibrosis score^69^ (0.82). When considering some studies exploiting AI techniques, we could verify that a new approach using LWA (learning by abstraction) method classifies liver ultrasound images as normal or abnormal and does not classify the data unless it is confident of accurate prediction. Features were extracted from ROIs within 99 ultrasound images and were used to train NN, SVM, and LWA classifiers with fivefold cross-validation. The proposed LWA method outperformed the other classifiers with an AUROC of 0.78^70^. In a second study, the prediction ability of particle swarm optimization (PSO), GA, MReg (multilinear regression), and alternative decision tree (ADT) algorithms were compared using medical data from 39,567 patients. Using uniform random sampling, the dataset was divided into training (22,690 patients) and test (16,877) sets. Four algorithms were applied for classification using tenfold cross-validation. The results evidenced that the ADT model had an AUROC between 0.73 and 0.76^71^. In another study factors provided by the 2005 updated ATP III clinical criteria for metabolic syndrome (MetS) along with age and gender were used to create a NAFLD prediction model. After preprocessing data from 40,637 patients they were divided into 66% and 34% for training and testing sets, respectively. The classification was performed by the J48 algorithm using hold-out cross-validation, and the AUROC of 0.731 was achieved^72^. NN also performed better in these cases.

From the described results, it can be seen that the NN, using AVI plus Glucose plus GGT plus Sex plus Age, produced few prediction errors in the test phase, whereas the accuracy percentage was not very high. However, the 18% error (18 of 100 subjects) in the test phase may be open to doubt, since it is possible that these subjects were developing NAFLD and so merely diagnosed in advance).

It has been demonstrated that the good performance of the ML algorithms used to identify NAFLD, applying common anthropometric parameters and other variables, can be a valid alternative to the classic indexes^73,74^.

Moreover, the NN was able to correctly identify all the subjects without NAFLD, as evidenced by the high VPP value (0.86). This VPP satisfies our objectives to detect subjects without NAFLD to avoid referral to perform more expensive diagnostic procedures.

This type of study highlights the fact that a NN can be used to find high-risk NAFLD subjects to send on for US. In this way, 82.6% of unnecessary US tests could be avoided (this value was calculated as the ratio of the total number of subjects in the web app test set, divided by the total number of subjects in the web app test set plus the number of false predictions).

In addition, to lighten the waiting lists, our aim was to develop a machine learning algorithm that would allow savings by eliminating a number of US that would otherwise be prescribed. The NN developed is therefore useful to exclude NAFLD and may be considered a valid diagnostic support in the context of epidemiological studies, not merely a smart working replacement diagnostic tool.

In conclusion, the NN can be considered a valid support for medical decision making in regard to health policies, in the context of epidemiological studies and screening.

### Study limitations

There are several limitations to this work. The most significant is that this study was conducted in a single center and so has a rather limited sample size. Deep learning models in other fields have included millions of samples. Another problem is that the NN is strongly linked to the identification of the NAFLD condition only in a Mediterranean population with the characteristics on which it was formed. A second limitation is the low sensitivity of the NAFLD diagnostic methodology, as it fails to detect a fatty liver content as low as > 25%^75^. However, both databases were drawn from population-based studies and subjects were selected from electoral lists or from the physicians lists. Moreover, participants subjects did not seek medical assistance and participated on a voluntary basis. Therefore, the NAFLD diagnosis performed by US was the only diagnostic procedure that could be proposed to participants, since biopsy or H-MRS would obviously be unethical.

### Future developments

In the future the NN based web app can be improved by using a SQL database where to save the entered data and, providing feedback to the app (correct or wrong prediction) in order to continue its training and make it more flexible so that it can be used on any kind of population. This could be done by leveraging a document classification system^76^ to retrieve data from electronic medical records and then building an open dataset^77^ in order to improve with more heterogeneous data the web app.

## Conclusion

The application of ML in the diagnosis of NAFLD is an efficient approach to identify healthy subjects. The model we propose has that can be exploited to target only those subjects who have a real need for further investigation, thus leading to a reduction in waiting lists, costs and time required for instrumental examinations. In this research we have predicted the risk of developing NAFLD in individuals using biochemical and anthropometric variables in a NN. The rationale behind our approach is divided into two parts: first train, evaluate performance and validate the result in assessing NAFLD risk in an individual. Second, development of a local web app that incorporates the previously evaluated NN, compare its performance applying in this way a rapid and non-invasive methodology in order to demonstrate that the proposed technique is suitable for optimal discrimination for NAFLD risk assessment. It is worthy to note that through XAI, it is possible to identify the factors that contribute to a given diagnosis. This facilitates the physician to do informed choices about their patients management and improve the health conditions of the subjects.

## Abbreviations

US: Ultrasound scan
NAFLD: Non-alcoholic fatty liver disease
WC: Waist circumference
HP: Hips
FLI: Fatty liver index
ML: Machine learning
AVI: Abdominal volume index
GGT: Gamma-glutamyl transferase
NASH: Non-alcoholic steatohepatitis
NN: Neural network
MRI: Magnetic resonance imaging
BP: Blood pressure
TP: True positive
TN: True negative
FP: False positive
FN: False negative
CSS: Cascading style sheets
HTML: HyperText markup language
AUC: Area under the curve
PPV: Positive predictive value
NPV: Negative predictive value
